# Fine Mapping of Gene Regions Regulating Neurodegeneration

**DOI:** 10.1371/journal.pone.0005906

**Published:** 2009-06-15

**Authors:** Maria Swanberg, Karin Harnesk, Mikael Ström, Margarita Diez, Olle Lidman, Fredrik Piehl

**Affiliations:** 1 Department of Clinical Neuroscience, Karolinska Institutet, Karolinska Hospital, Stockholm, Sweden; 2 Department of Clinical Sciences, Malmö, Lund University, Malmö, Sweden; Universidade Federal do Rio de Janeiro (UFRJ), Instituto de Biofísica da UFRJ, Brazil

## Abstract

**Background:**

Damage to nerve cells and axons leading to neurodegeneration is a characteristic feature of many neurological diseases. The degree of genetic influence on susceptibility to axotomy-induced neuronal death has so far been unknown. We have examined two gene regions, *Vra1* and *Vra2,* previously linked to nerve cell loss after ventral root avulsion in a rat F2 intercross between the DA and PVG inbred rat strains.

**Methodology/Principal Findings:**

In this study, we use two generations (G8 and G10 cohorts) of an advanced intercross line between DA and PVG^av1^ to reproduce linkage to *Vra1* and to fine-map this region. By isolating the effect from *Vra1* in congenic strains, we demonstrate that *Vra1* significantly regulates the loss of motoneurons after avulsion. The regulatory effect mediated by *Vra1* thus resides in a congenic fragment of 9 megabases. Furthermore, we have used the advanced intercross lines to give more support to *Vra2*, originally detected as a suggestive QTL.

**Conclusions/Significance:**

The results demonstrated here show that naturally occurring allelic variations affect susceptibility to axotomy-induced nerve cell death. *Vra1* and *Vra2* represent the first quantitative trait loci regulating this phenotype that are characterized and fine mapped in an advanced intercross line. In addition, congenic strains provide experimental evidence for the *Vra1* effect on the extent of injury-induced neurodegeneration. Identification of the underlying genetic variations will increase our understanding of the regulation and mechanisms of neurodegeneration.

## Introduction

Many diseases of the central nervous system (CNS) are characterized by neuron/axon damage leading to neurodegeneration. The complex aetiology of neurodegenerative disorders includes a genetic predisposition. Unlike monogenic traits, most genes that are involved in common complex diseases are likely to be evolutionarily conserved, vary between diseased individuals, and only modestly affect risk. This makes disease predisposing genes difficult to identify, and so far only a small number of genes regulating complex traits have been characterized. One strategy is to genetically dissect disease phenotypes in intercrosses of inbred rodent strains, which are susceptible or resistant in relevant disease models [1], [2], [3]. Genome wide linkage analysis can be performed in an F2 intercross between two inbred strains. All F2 individuals are related but genetically unique, which enables positioning of quantitative trait loci (QTLs). Further intercrossing generates an advanced intercross line (AIL), which increases the genetic resolution and allows for fine mapping of QTLs identified in a genome wide linkage analysis. This has proven fruitful for resolving the genetic contribution to complex traits such as autoimmune neuroinflammation, reviewed in [4]. Apart from identifying candidate genes that can be tested in clinical materials, this type of experimental genetic dissection can unravel information about disease-related molecular pathways [3], [5], [6].

We have set out to characterize the genetic influence on neurodegeneration in a simple and reproducible mechanical nerve lesion model; ventral root avulsion (VRA) in the rat [7], [8], [9]. VRA results in a very proximal axotomy of motor axons at the boundary of the central and peripheral nervous systems, with a subsequent substantial loss of axotomized cells during the second and third post-operative weeks. Lesioned motoneurons deprived of physical contact with peripheral nerve tissue, thus, degenerate in a similar fashion as many other CNS nerve cell populations [10]. This makes VRA a useful model for studying neurodegeneration in the CNS. Previously we reported results from a whole genome scan of an F2 intercross between the DA and PVG (RT1C) rat strains, which revealed four QTLs regulating different aspects of the VRA response. *Vra1* and *Vra2* were linked to neurodegeneration, *Vra3* displayed suggestive linkage to T cell infiltration and *Vra4* was linked to differential expression of MHC class II [11]. Fine mapping of *Vra4* in an AIL between DA and PVG^av1^ rats, combined with expression studies and gene sequencing in a set of inbred strains, resulted in the identification of the underlying gene, *Mhc2ta*, encoding the MHC class II transactivator. This gene was also found to be associated to human inflammatory disorders [5]. In this study we set out to reproduce and fine map the strongest QTL, *Vra1,* with regard to neurodegeneration by studying two generations of the DA and PVG^av1^ AIL and *Vra1* congenic animals. This refines a 9 megabases (Mb) large fragment that significantly regulates motoneuron loss after VRA. Further, we use the experimental setup of the AIL to give more support to and fine map the second suggestive QTL, *Vra2*. Taken together, our data demonstrate a substantial genetic regulation of nerve-injury induced neurodegeneration and provide information about two candidate gene regions, one of them with high genomic resolution.

## Results

### Fine mapping of *Vra1* using an advanced intercross line

**Figure 1:**
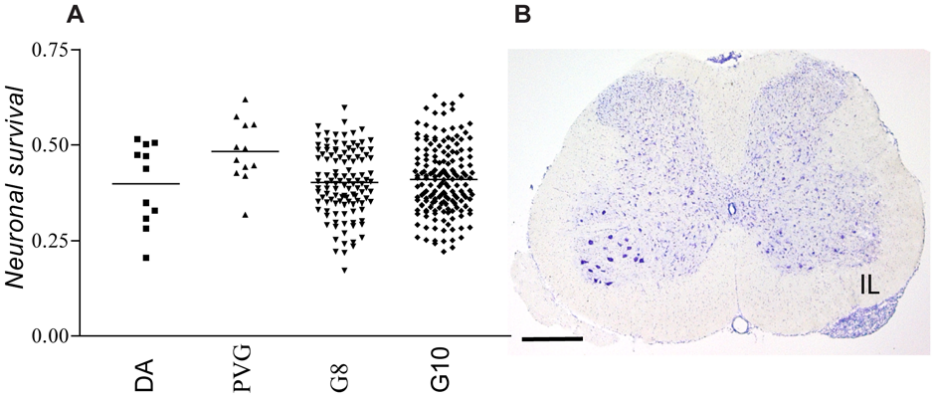
Motoneuron survival after ventral root avulsion (VRA). (A) Distribution of nerve cell survival in parental controls and AIL cohorts, sampled 14 days after VRA. Horizontal lines indicate mean survival rate for DA (0.38), PVG^av1^ (0.47), G8 (0.40), G10 (0.41). (B) Cresyl violet counter stained section demonstrating loss of motoneurons on the side of the lesion (IL) in DA rat 21 days after VRA (IL = ipsilateral), scale bar = 0.5 mm.

Two generations, the G8 and G10 of a DAxPVG^ av1^ AIL were used for finemapping of *Vra1* and *Vra2*. The G8 and G10 cohorts consisted of 126 and 186 males, respectively. Thirteen and ten animals in the G8 and G10 cohorts, respectively, were excluded from the analysis due to incomplete lesion. The success rate for genotyping was 97.9% in G8 and 97.6% in G10. Nerve cell counts in the parental control strain demonstrated 47% and 38% mean nerve cell survival in PVG^av1^ and DA strains, respectively, corresponding to a 23% relative increase in survival in PVG^av1^ compared to DA. The mean survival in the G8 and G10 cohorts 14 days after VRA was 40% and 41%, respectively (Fig 1A). A cresyl violet stained section, demonstrating neuron loss in the ventral cord in the DA rat 21 days after VRA, is shown in Fig 1B.

**Figure 2:**
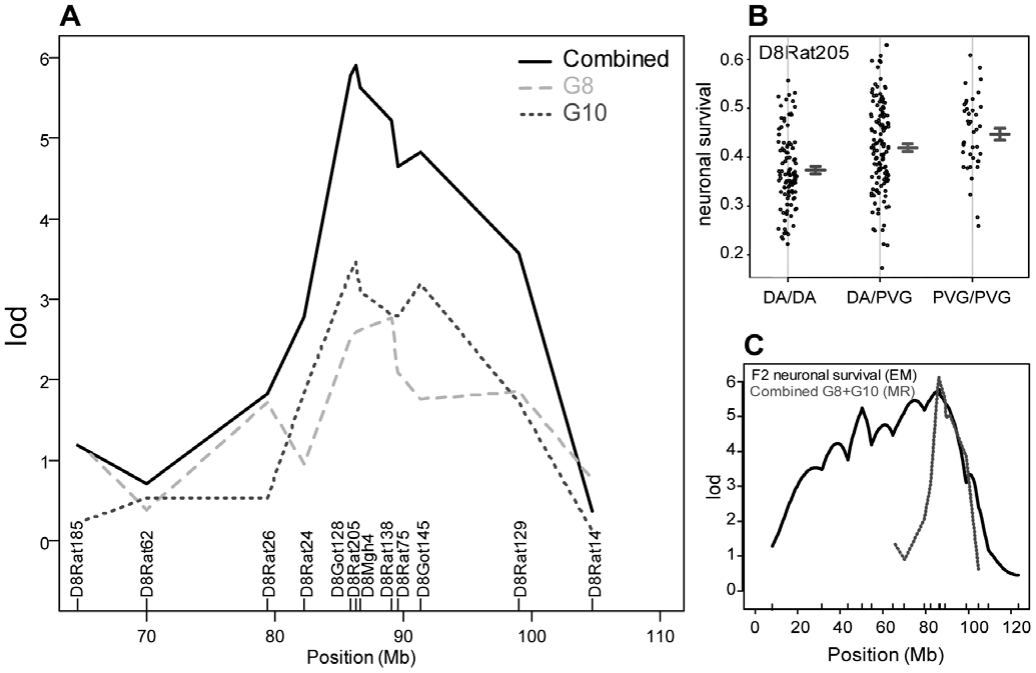
Genetic linkage to neurodegeneration for the *Vra1* region. (A) LOD score plot for linkage to *Vra1* in the individual G8 and G10 cohorts, and meta-analysis calculated by marker regression for the combined dataset. (B) Survival of motoneurons in the combined population stratified for genotype at the max marker of *Vra1* (D8Rat205), demonstrating reduced survival in rats homozygous for the DA allele. Lines indicate the mean with error bars at +/− 1 SE. (C) Comparison of the original LOD score plot obtained in the F2 population and the current obtained in the AIL.

*Vra1* was covered by 19 markers spanning 54 Mb in the G8 cohort and 13 markers spanning 40 Mb in the G10 cohort. *Vra1* was linked to the degree of motoneuron loss in both AIL cohorts, thus replicating the linkage originally described in the F2 intercross. The logarithm of odds (LOD) scores were 3.46 and 3.19 for the G8 and G10 cohorts, respectively, using the multiple imputation method (Fig 2A). This exceeds the threshold levels obtained by calculating family residual effects, which were 1.19 and 1.39, and the 95% experiment-wise threshold level of 2.79 and 2.60 in G8 and G10 data sets, respectively, as generated by permutations. Furthermore, a combined cross analysis based on 12 common markers in the G8 and G10 cohorts resulted in strengthened evidence for linkage to neurodegeneration for *Vra1*, as the LOD score rose to 5.9 using marker regression analysis (Fig 2A). The mean cell survival of animals that were homozygous (DA/DA or PVG^ av1^/PVG^ av1^) or heterozygous (DA/PVG^ av1^) at the max marker D8Rat205 differ significantly (Fig 2B). In addition, the AIL experiments narrowed the candidate region from 55 Mb in the F2 cross to roughly 16 Mb (Fig 2C). Data on flanking and max markers, genomic position and confidence intervals for *Vra1* in the G8, G10 and combined linkage peaks are presented in Table 1.

**Table 1:** Summary of max and flanking microsatellite markers, and LOD scores for the different linkage peaks obtained in G8, G10 and the combined cross analysis, respectively.

Method	*G8*		*G10*			*Combined*		
						*G8*	*G10*	*G8+G10*
	Imputation		Imputation			Marker regression	Marker regression	Marker regression
QTL	VRA1	VRA2	VRA1	VRA2	VRA1/VRA2ADDITIVE	VRA1	VRA1	VRA1
Peak marker(s) (Position, Mb)	D8Rat138 (89.06)	N/A	D8Mgh4 (86.63)	D5Rat190 (24.98)	D5Rat190/D8Rat75 (24.98,89.56)	D8Rat138 (89.06)	D8Rat205 (86.29)	D8Rat205 (86.29)
LOD score	3.46	N/A	3.19	2.39	6.39	2.77	3.45	5.87
1.8 LOD drop interval (Position, Mb)	D8Rat26-D8Rat14 (79.35–104.7)	N/A	D8Rat26-D8Rat14 (79.35–104.7)	D5Rat209-D5Rat6 (23.24–52.53)	N/A	D8Rat185-D8Rat14 (64.57–104.7)	D8Rat26-D8Rat14 (79.35–104.7)	D8Rat24-D8Rat129 (82.25–98.95)
99% Bayesian credible interval (Position, Mb)	D8Rat38-D8Rat14 (54.63–104.7)	N/A	D8Rat26-D8Rat14 (79.35–104.7)	D5Rat209-D5Rat6 (23.24–52.53)	N/A	D8Rat185-D8Rat129 (64.57–98.95)	D8Rat24-D8Rat129 (82.25–98.95)	D8Got128-D8Rat129 (85.83–98.95)

### Reproduction of *Vra2*

**Figure 3:**
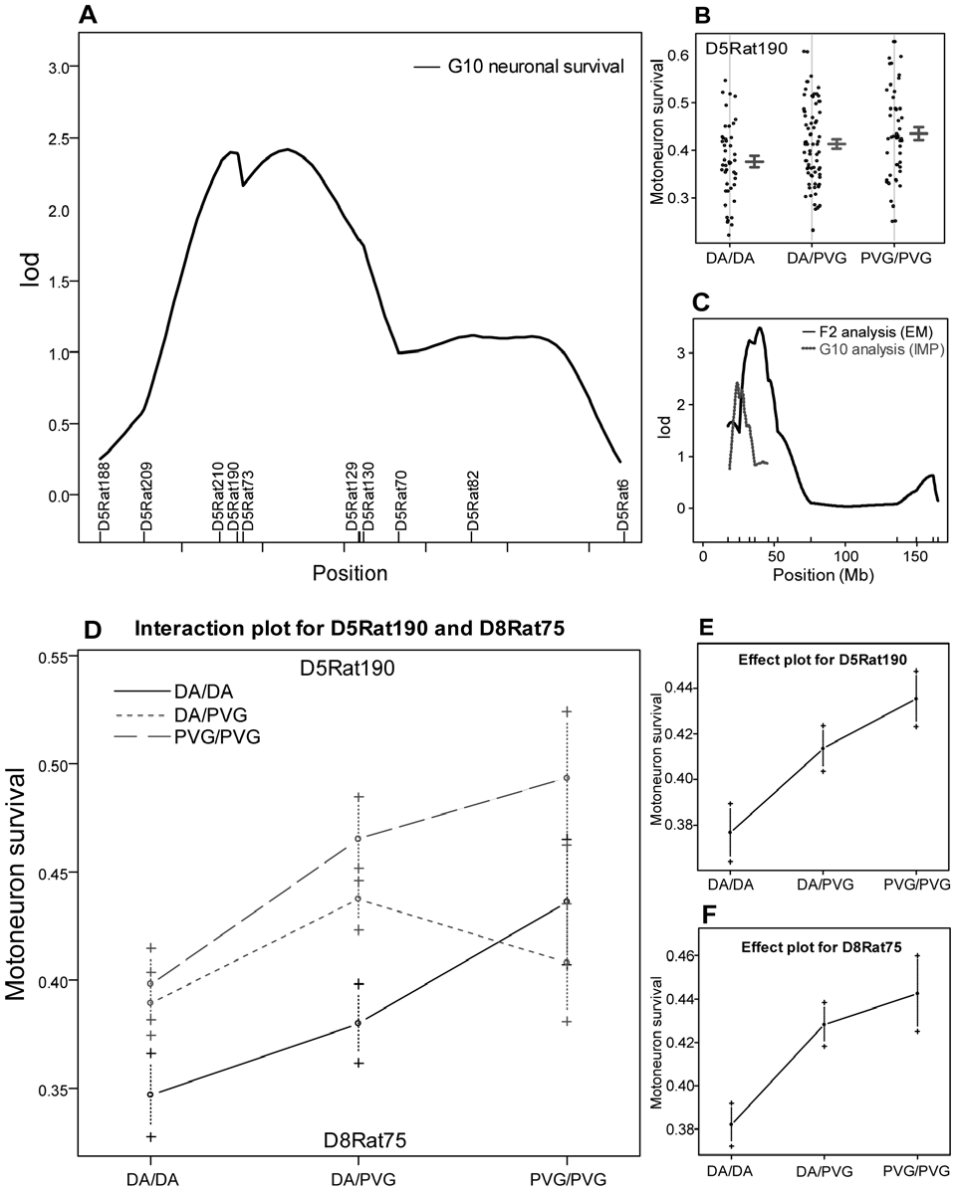
Genetic linkage to neurodegeneration for the *Vra2* region on chromosome 5. (A) LOD score plot for linkage to *Vra2* in the G10 cohort. Survival of motoneurons in the G10 (B) population stratified for genotype at the max marker of *Vra2* (D5Rat190), demonstrating reduced survival in rats homozygous for the DA allele. Lines indicate the mean with error bars at +/− 1 SE. (C) Comparison of the original LOD score plot obtained in the F2 population with the current obtained in the AIL. (D) An interaction plot shows an additive effect in terms of increased nerve cell loss in carriers of DA alleles both at the *Vra1* and *Vra2* positions and the lowest nerve cell loss for individuals being PVG^av1^ homozygous at both loci. Animals homozygous at both positions have similar survival ratios as the corresponding parental controls. Effect plot of markers giving max additive LOD for *Vra2* (E) and *Vra1* (F) in the G10 cohort.

A second gene region, *Vra2* on chromosome 5, was identified as a QTL displaying suggestive linkage to nerve cell death in the original F2 intercross [11]. To cover the *Vra2* F2 linkage peak, 13 markers spanning 40 Mb were used in the G8 cohort, and 12 markers spanning 34 Mb were used in the G10 cohort. While no evidence of linkage to the *Vra2* region was found in G8, linkage with a LOD score of 2.39 for the max marker (D5Rat190) was found in the G10 cohort (Fig 3A). This value is slightly lower than that of the F2 population (Fig 3C), but exceeds the family effect level of 1.35 and the 90% experiment-wise threshold level of 2.29. The linkage to *Vra2* in the G10 cohort thereby reach the same level of significance as in the original F2 cross. The average cell survival of animals carrying DA or PVG^av1^ alleles at D5Rat190 is illustrated in Fig 3B. Combined analysis of G8 and G10 for *Vra2* did not increase linkage and only reached significance when cross was used as covariate, showing that the effect is cross specific for the G10 cohort.

### *Vra1-Vra2* interaction analysis

A two dimensional scan in the G10 cohort revealed a strong additive effect of *Vra1* and *Vra2,* with a LOD score of 6.39 using the imputation method. The maximum additive LOD score was detected with the markers D5Rat190 and D8Rat75 (see Table 1). In order to check whether the two analyzed QTLs separately contributed to the LOD score, the multiple QTL model was fitted to allow for the dropping of a single QTL. The dropping of *Vra1* or *Vra2* showed a highly significant reduction of the additive LOD (p<0.001 with F statistics). In addition, the interaction plot shows an increased nerve cell loss in carriers of DA alleles at both the *Vra1* and *Vra2* positions (Fig 3D), which further supports the existence of an additive effect between these two loci. The effect plots of the markers displaying the maximum LOD score for *Vra1* and *Vra2* are shown in Fig 3E and 3F, respectively. Animals homozygous for DA alleles at both max markers display a 35% mean nerve cell survival as compared to 49% mean survival in animals homozygous for PVG^av1^ alleles at both markers. This can be compared to the mean values of 38% and 47% for parental DA and PVG^av1^ strains, respectively. This suggests that the additive effect of *Vra1* and *Vra2* accounts for a large part of the difference observed between the two parental strains.

### DA.PVG-*Vra1* congenic rat strains

**Figure 4:**
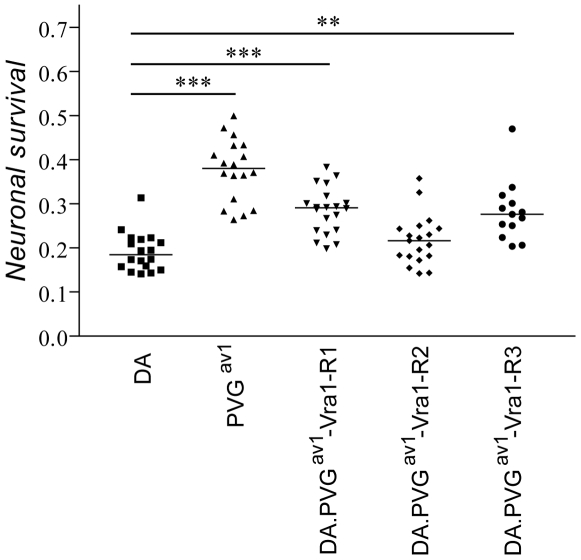
Survival of motoneurons 21 days after VRA in congenic rats containing overlapping genomic fragments of the *Vra1* region. The results are presented as pooled data from two independent experiments, which yielded highly similar results. Motoneuron survival in R1 and R3 animals is significantly increased compared to DA controls, whereas R2 animals retain the DA phenotype. PVG^av1^ parental animals are also included as a measure of the parental strain differences. Lines indicate the median. (**p<0.01, ***p<0.001).

The *in vivo* effect of *Vra1* was examined in three separate congenic strains that contain overlapping fragments of the *Vra1* region from the PVG^av1^ genome on DA background: DA.PVG^av1^-*Vra1-*R1 (R1), DA.PVG^av1^-*Vra1-*R2 (R2) and DA.PVG^av1^-*Vra1-*R3 (R3). In a first experiment, survival of motoneurons 21 days after VRA in congenic animals was compared to parental DA controls, showing a significantly increased survival of axotomized cells in R1 (p<0.05), but not R2 animals. The experiment was repeated with the R3 strain included. Again, motoneuron survival in the R1 strain was significantly higher 21 days after VRA compared to DA parental controls (p<0.05). The R3 strain also displayed a significant reduction of nerve cell loss compared to the DA strain (p<0.01). As intra-strain differences in mean survival ratios and standard deviations are very small between the two experiments, results are presented as pooled data (Fig 4). Pooling the data resulted in an even more significant effect of the R1 fragment, with a mean nerve cell survival of 28% in the congenic strain representing a 48% increase compared to the parental strain control (DA; p<0.001). Motoneuron survival in R3 was identical to the R1 strain (28%; p<0.01 compared to DA). R2 did not differ from control DA animals (22% and 19%, respectively).

**Figure 5:**
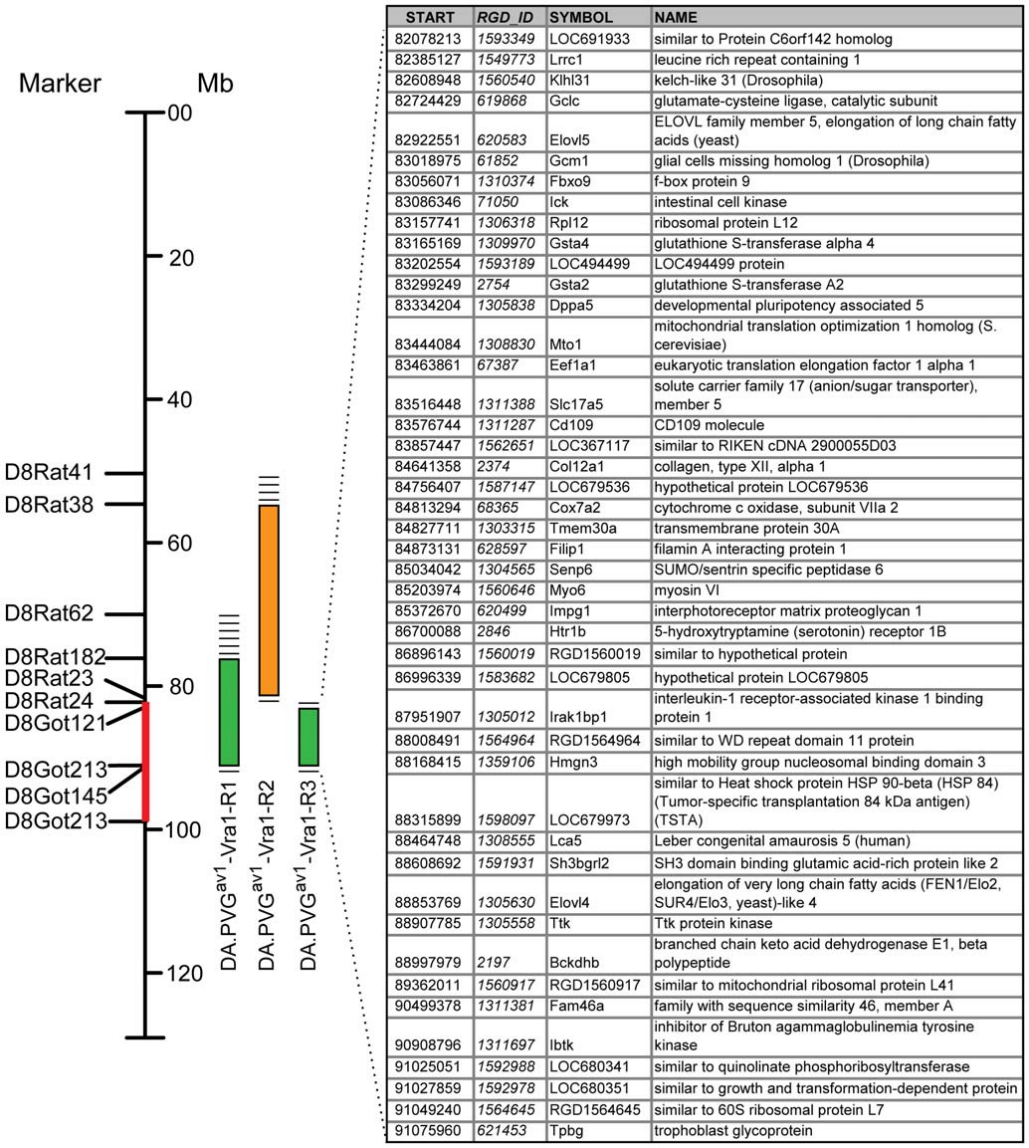
View of chromosome 8 with congenic fragments of DA.PVGav1-*Vra1-*R1, -R2 and -R3 strains indicated on the left and the 1.8 LOD drop interval for G8+G10 combined QTL analysis marked in red. The 45 protein coding genes located in the shared congenic fragments of R1 and R3animals are listed on the right. Dashed lines indicate regions between microsatellite markerswhere the recombination has occurred, thereby being genes possibly included in the fragment. Information about genes is based on Rat Genome V3.4 Assembly.

The mean nerve cell survival at 21 days was 19% in parental DA rats as compared to 38% in parental PVG^av1^ rats (p<0.001). In the AIL experiment, the difference between the parental strains was less pronounced, which is explained by the later time-point used for determining motoneuron survival in the congenic study. The outcome of the experiment strongly suggests that the causative genetic variation of *Vra1* is located within the common genomic region of the R1 and R3 strains, but outside of the R2 fragment, i.e. a 9 Mb genomic interval between the markers D8Rat24 and D8Got145. A schematic map of the congenic fragments' location on chromosome 8, including the genes in the interval, is depicted in Figure 5.

Comparative genomics revealed 12 QTLs in the synthenic mouse region (chr 9 approx 78–90 Mb), none of which were linked to neurodegenerative disorders (data not shown). However, this probably reflects the fact that few, if any, studies attempting to map QTLs for phenotypes related to neurodegeneration have been published. Further, 42 human homologues for the rat genes in the R3 fragment were found,of which most are located on chromosome 6. Mutations in a number of the human genes cause various Mendelian disorders, but only one of them shows association to complex disease in Genatlas (www.genatlas.org). Human homologues with associated diseases are presented in Supporting Table S1.

## Discussion

We here demonstrate a considerable variability in susceptibility to nerve injury-induced neurodegeneration that can be attributed to naturally occurring genetic differences between the inbred DA and PVG^av1^ strains.

Loss of nerve cells is a characteristic feature of many CNS diseases. It may be secondary to traumatic injuries or inflammatory diseases such as multiple sclerosis (MS), or occur as a primary disease process, as is the case in amyotrophic lateral sclerosis (ALS) and Parkinson's disease. The common forms of these diseases often have a complex etiology, which includes a genetic component [12]. Knowledge about disease predisposing genes is of importance for disclosing pathogenic pathways that could be targeted therapeutically. However, unlike monogenic diseases, genes involved in complex traits often only modestly affect disease and are therefore more difficult to identify. So far, very few studies have examined the genetic impact on neurodegenerative processes using an unbiased experimental approach [13], [14], [15].

The two regions examined here, *Vra1* and *Vra2,* are the first nerve cell death regulating QTLs to be characterized and fine mapped using an advanced intercross line. These findings suggest that genetic variability in this key feature of the axon reaction is controlled by a low number of gene loci containing genes that are polymorphic between the examined strains. We report independent observations of significant effects of *Vra1* on the degree of neurodegeneration in genetically heterogenous intercrossed animals as well as in congenic strains, where only the *Vra1* locus has been exchanged between the two parental strains. This lends strong support for functional biological effects originating from genetic elements within the *Vra1* locus. In the preceding F2 scan, the approximate physical genomic size of *Vra1* locus was 55 Mb. The confidence interval of the combined AIL cohorts now reduce the size to approximately 16 Mb. In addition, the congenic experiment provides further reduction of the candidate interval to just over 9 Mb. Preserved phenotypic effect of a QTL in a congenic strain is the ultimate proof of linkage. In this context, the *Vra1* region is unique in showing replicated significant effects on motoneuron survival after nerve injury.

In the F2 scan, Vra2 displayed suggestive linkage to neuronal loss after VRA. In this study, we reproduce suggestive linkage of neurodegeneration to *Vra2* in the G10, but not the G8 cohort. Nevertheless, in the G10 cohort both *Vra1* and *Vra2* show independent linkage as well as a highly significant contribution to the combined linkage, which supports a biological effect of *Vra2*. Furthermore, individuals which are homozygous at both loci for either of the parental genotypes almost exactly resemble the outcome in the corresponding parental control. These two QTLs taken together thus account for a major part of the difference between the two parental strains.

After nerve avulsion, neurodegeneration is initiated around 7 days after injury and reaches a plateau at day 21 [8]. The AIL populations were sampled 14 days after injury, to comply with analyses of other aspects of the VRA response, whereas the congenic animals were sampled at day 21. Thus, the QTLs detected in the intercross lines are mapped at a timepoint when only half of the degeneration has occurred, which may reduce power to detect the relatively weaker linkage to *Vra2*. Studies in animals congenic for *Vra2* at 21 days after injury will likely increase the possibility to capture the effect of this locus as the segregation between parental strains with regard to neurodegeneration increases with time after injury. Congenic strains are now being bred in our laboratory, to allow for functional verification of *Vra2*.

The *Vra1* candidate region of 9 Mb contains 59 genes in the rat of which 45 are protein coding and 14 pseudo genes, which is too large a number to pinpoint candidate genes. However, the list contains a number of genes that are of importance for cell cycle regulation, a process which recently has been implicated in neurodegeneration (see eg.[16], [17], [18]), and genes encoding reducing agents regulating oxidative stress, which is implicated in neurodegenerative disorders such as Alzheimer's disease, Parkinson's disease and ALS [19], [20], [21].

However, comparative genomics revealed no known association of the human homologues of the *Vra1* genes to any human complex disease, underscoring the need for further investigation of this region.

In conclusion, we here genetically characterize the first two regions that regulate axotomy-induced nerve cell deaths and provide a comprehensive list of candidate genes in the *Vra1* interval. Most importantly, we provide experimental, biological evidence from *Vra1* congenic rat strains that *Vra1* regulates the degree of motoneuron degeneration after nerve injury. We also give further support to the suggestive QTL *Vra2* and show a significant additive effect of these two loci. Further studies are ongoing to exactly define the underlying genetic variation underlying *Vra1,* primarily using recombinant breeding of existing congenic strains. Positional identification of responsible genes and the use of comparative genomics will be of importance for the understanding of the genetic regulation of susceptibility to neurodegeneration and may increase our understanding of conditions such as brain and spinal cord trauma, MS, cerebrovascular and neurodegenerative diseases.

## Materials and Methods

### Animals and breeding

The DA (RT1AV1) strain was originally generously provided by Professor Hans Hedrich (Medizinische Hochschule, Hannover, Germany), while the PVG-RT1AV1 (hereafter called PVG^av1^) strain was obtained from Harlan UK Ltd (Blackthorn, UK). These two rat strains carry the same MHC haplotype and any phenotypic differences in crosses between them will originate from non-MHC genes. Animals used for experiments were bred in our in-house breeding facility under specific pathogen-free and climate-controlled conditions with 12 h light/dark cycles, housed in polystyrene cages containing wood shavings, and fed standard rodent chow and water *ad libitum*. The AIL was created by reciprocal breeding of DA and PVG^av1^ rats. Subsequent crossings of offspring from 50 breeding pairs were carried out for eight (G8) or ten (G10) generations, respectively. In a previous F2 scan parental animals displayed a 36% relative increase in survival of motoneurons 14 days following VRA in PVG rats compared to DA [11]. Although mean survival rates of motoneurons did not differ between males and females, the variability obtained in the F2 cohort tended to be greater in females and linkage to *Vra1* and *Vra2* was weaker. Therefore, only males were studied in the experiments presented here. VRA experiments were performed in two AIL populations, G8 and G10. In the G8 generation, 12 rats from each parental strain and 126 male rats were used and in the G10 generation, 12 DA rats and 11 PVG^av1^ parental rats and 186 male rats.

The congenics were bred by selecting male PVG^av1^ allele donors from the G8 AIL cohort, in order to transfer relatively short and well defined overlapping genome fragments of the *Vra1* region. The congenic intervals are defined by the following excluding markers, DA.PVG^av1^-*Vra1-*R1 (RNO8: D8Rat146-D8Got145, [73.1–91.3 Mb]); DA.PVG^av1^-*Vra1-*R2 (RNO8: D8Rat41-D8Rat24 [50.4–82.2 Mb]). Repeated backcrossing to the recipient DA strain was performed for an additional nine generations to create congenics with theoretically <0.1% of the donor genome outside the *Vra1* locus. The DA.PVG^av1^-*Vra1-*R3 (RNO8: D8Rat24-D8Got145 [82.2–91.3 Mb]) is a recombinant from the DA.PVG^av1^-*Vra1-*R1 congenic and was bred separately after a recombination event in the 4^th^ generation of backcrossing to DA. Experiments on congenic rats were performed with 9–10 animals in each group.

### Nerve lesion

All animals were subjected to unilateral avulsion of the left L3-L5 ventral roots under standardized conditions and in deep isoflurane anesthesia at an age of 6–8 (G8) or 8–10 (G10 and congenics) weeks with a post-operative survival time of 14 (G8 and G10) or 21 (congenics) days (+/− 2 hrs), after which animals were killed with CO_2_ and perfused with cold PBS. The shorter post-operative survival time in the AIL cohorts was chosen to comply with the survival time used in the original F2 genome-wide scan [11]. Data on genetic differences in VRA-induced expression of MHC class II in the G8 and a subset of animals from the G10 cohort has been presented previously [5]. In the congenics experiment a three weeks post-operative survival was chosen, since parental strains differ more with regard to nerve cell survival at this time point [8]. After dissection the spinal cords were carefully examined in a dissection microscope to verify the completeness of the lesion. In no case did the microscopic examination reveal signs of hemorrhage, necrotic zones or direct damage to the cord. Animals with incomplete lesions were excluded from further analysis. All experiments in this study were approved by the local ethical committee for animal experimentation (Stockholms Norra Djurförsöksetiska Nämnd) and comply with the guidelines from the Swedish National Board for Laboratory Animals and the European Community Council Directive (86/609/EEC).

### Cell counts

Serial transverse frozen sections (14 µm) of tissue from the L4 segment of the spinal cord were cut with a cryostat. Nerve cell counts of cresyl violet counterstained sections were performed as described previously [9], [11]. In brief, counts of motoneurons with a visible nucleus were performed blindly by a single observer on every fifth section with a total of 20 sections from each rat, in total covering a distance of approximately 2 mm in the rostro-caudal axis. Each slide contained sections from 9 rats mounted in a similar fashion in order to reduce the risk of bias. Parental rats were randomly included as a measure of inter-individual consistency. G8, G10 and congenic rat cohorts were counted by the same person, but at separate occasions. In a previous study the reduction in mean soma size of axotomized cells as compared to unlesioned cells on the contralateral side was highly similar (−27–28%) regardless of sex or strain [11]. No correction for cell shrinkage was performed here as this feature is expected to affect the studied cell populations to a similar degree. The degree of neurodegeneration is presented as a ratio of the total number of motoneurons on lesioned and unlesioned sides, respectively, in each rat. Micrographs were recorded on a Zeiss Axioskop microscope system.

### Genotyping

Genomic DNA was extracted from tail tips or ear clippings using a standard protocol. PCR-primers for polymorphic simple sequence length polymorphisms (SSLPs) were selected from available internet databases (Rat Genome Database (rgd.mcw.edu), Center for Genomic Research, Whitehead Institute/MIT (www-genome.wi.mit.edu/rat/public), Ensembl (www.ensembl.org) and UniSTS at NCBI (www.ncbi.nlm.nih.gov). The primers were purchased from PROLIGO (Paris, France). One primer in each pair was labeled with [γ-^33^P]ATP (PerkinElmer, Boston, MA), genomic DNA was amplified with a standard PCR protocol, and the amplified fragments were separated on 6% polyacrylamide gels (National Diagnostics, Atlanta, GA). Genotypes were recorded manually from autoradiographic films (Biomax, Kodak, Rochester, NY) independently by two investigators. DNA from DA and PVG^av1^ rats were included for each marker.

### Linkage analysis and statistics

Linkage analysis was performed using GNU R 2.6.0 with the QTL package version 1.07–12 (R/qtl) [22]. Analysis was performed for the G8 and G10 generations separately but also in a combined analysis to increase statistical power. Separate analyses were performed with the multiple imputation method (normal model) with 64 simulations (step = 2, ndraws = 64) in R/qtl. Because different generations were combined in the combined cross analysis, marker regression with 64 simulations was used. Only physical positions for the markers were used in the combined analysis since they, in contrast to genetic positions, are constant between generations. Experiment-wise significance threshold levels were generated by the permutation method in R/qtl using 10 000 permutations [23]. Furthermore, family residuals were calculated to rule out that peaks are derived from family effects. This was done by subtracting the average litter phenotype score from that of each individual sibling in that litter, and running the same imputation analysis as above on the data obtained. The highest LOD score from this analysis was used as the threshold level for family effect. Confidence intervals for the peaks were determined by using 1.8 LOD support intervals and 99% Bayesian credible intervals [24]. A two-dimensional scan was performed for interactions and additive influences. A multiple qtl model was fitted dropping the effect of one of the QTLs at a time to determine interaction and significance for each of the QTLs on the full model. LOD-plots and effect/interaction-plots were generated with R/qtl.

Significance levels for phenotypic differences between the parental and congenic strains were calculated with non-parametric ANOVA, with Dunn's Multiple Comparisons Test, using GraphPad Prism 3.0 (GraphPad Software Inc, San Diego, CA).

Comparative genomics was used to check the smallest congenic fragment for synthenic human and mouse regions with Ensembl release 53. All human homologue genes were checked in Genatlas (release Tue 30 Dec 2008), and the mouse synthenic region was checked in the MGI_4.2 database, for association and/or linkage to disease.

## Supporting Information

Table S1 Human genes, homologous with genes in smallest congenic fragment on rat chromosome 8. Diseases associated with the human genes are given in the right column. List of human homologues to the rat genes located within the R3 congenic fragment. No association of any human gene to complex neurodegenerative disease.
(0.11 MB DOC)
