# Supplementary material for: Fine Mapping of Gene Regions Regulating Neurodegeneration
Source: PLoS One. 2009 Jun 15;4(6):e5906. doi: 10.1371/journal.pone.0005906 (PMC2691596; doi:10.1371/journal.pone.0005906)
Supplement: Table S1 — Human genes, homologous with genes in smallest congenic fragment on rat chromosome 8. Diseases associated with the human genes are given in the right column. List of human homologues to the rat genes located within the R3 congenic fragment. No association of any human gene to complex neurodegenerative disease. (0.11 MB DOC) [file pone.0005906.s001.doc]

| **Rat gene** | | **Human homologue** | | **Human disease association** |
| --- | --- | --- | --- | --- |
| **ID** | **Position** | **ID** | **Position** |  |
| LOC681849 (ENSRNOG00000005934) | 8:82108897-82200531 |  |  |  |
| ENSRNOG00000037691 | 8:82333330-82333845 |  |  |  |
| LOC367113 (ENSRNOG00000005970) | 8:82385127-82498041 | LRRC1 (ENSG00000137269) | 6:53767737-53896878 |  |
| NP_001101640.1 (ENSRNOG00000006224) | 8:82624289-82629245 | KLHL31 (ENSG00000124743) | 6:53620658-53638465 |  |
| Gclc (ENSRNOG00000006302) | 8:82724429-82762850 | GCLC (ENSG00000001084) | 6:53470098-53517790 | association of polymorphism(-129C/T) with myocardial infarction |
| Elovl5 (ENSRNOG00000006331) | 8:82922551-82948348 | ELOVL5 (ENSG00000012660) | 6:53240155-53321901 |  |
| Gcm1 (ENSRNOG00000007932) | 8:83018975-83032211 | GCM1 (ENSG00000137270) | 6:53099721-53121586 |  |
| Fbxo9 (ENSRNOG00000008214) | 8:83056071-83077586 | FBXO9 (ENSG00000112146) | 6:53037755-53073630 |  |
| Ick (ENSRNOG00000008691) | 8:83086346-83140977 | ICK (ENSG00000112144) | 6:52974068-53034559 | endocrine-cerebro-osteodysplasia, autosomal recessive |
| Rpl12_predicted (ENSRNOG00000002211) | 8:83157769-83158495 |  |  |  |
| LOC687840 (ENSRNOG00000030449) | 8:83165170-83183054 |  |  |  |
| LOC494499 (ENSRNOG00000029861) | 8:83202554-83211997 | GSTA3 (ENSG00000174156) | 6:52869396-52882455 |  |
|  |  | GSTA1 (ENSG00000187919) | 6:52764374-52776616 | Over-expressed in proliferative inflammatory atrophy |
|  |  | GSTA2 (ENSG00000096087) | 6:52722898-52736232 |  |
| LOC494499 (ENSRNOG00000029861) | 8:83299026-83315635 | GSTA5 (ENSG00000182793) | 6:52804412-52818945 |  |
| Gsta2 (ENSRNOG00000000201) | 8:83299026-83315635 | GSTA3 (ENSG00000174156) | 6:52869396-52882455 |  |
|  |  | GSTA1 (ENSG00000187919) | 6:52764374-52776616 |  |
|  |  | GSTA2 (ENSG00000096087) | 6:52722898-52736232 |  |
|  |  | GSTA5 (ENSG00000182793) | 6:52804412-52818945 |  |
| Dppa5_predicted (ENSRNOG00000000199) | 8:83334204-83335056 | DPPA5 (ENSG00000203909) | 6:74119508-74120674 |  |
| RGD1311103_predicted (ENSRNOG00000025957) | 8:83342414-83343431 | OOEP (ENSG00000203907) | 6:74134997-74161537 |  |
| NP_001100311.1 (ENSRNOG00000037659) | 8:83444223-83456934 |  |  |  |
| Eef1a1 (ENSRNOG00000009439) | 8:83463585-83466803 |  |  |  |
| Slc17a5 (ENSRNOG00000009330) | 8:83516440-83551409 | SLC17A5 (ENSG00000119899) | 6:74359824-74420418 | Autosomal recessive mutation causes Sialic acid storage disease, Salla disease |
| Cd109 (ENSRNOG00000025332) | 8:83575190-83672770 | CD109 (ENSG00000156535) | 6:74462235-74594761 |  |
| LOC367117 (ENSRNOG00000031132) | 8:83857447-83862539 | MRPL42 (ENSG00000198015) | 12:92385401-92420568 |  |
| ENSRNOG00000029176 (ENSRNOG00000029176) | 8:84414019-84414282 | AL136040.5 (ENSG00000205579) | 14:80782391-80782660 |  |
| ENSRNOG00000025186 (ENSRNOG00000025186) | 8:84606853-84607621 |  |  |  |
| Col12a1 (ENSRNOG00000010510) | 8:84641359-84756923 | COL12A1 (ENSG00000111799) | 6:75850762-75972343 |  |
| Q6TXH7_RAT (ENSRNOG00000010657) | 8:84809074-84817572 |  |  |  |
| Tmem30a (ENSRNOG00000010895) | 8:84827711-84849715 | TMEM30A (ENSG00000112697) | 6:76019369-76051221 |  |
| Filip1 (ENSRNOG00000011521) | 8:84873131-84902872 | FILIP1 (ENSG00000118407) | 6:76062346-76260216 |  |
| NP_001100312.1 (ENSRNOG00000024336) | 8:85123608-85183953 | SENP6 (ENSG00000112701) | 6:76368320-76484717 |  |
| RGD1560646 (ENSRNOG00000011852) | 8:85203974-85368017 | MYO6 (ENSG00000196586) | 6:76515629-76685974 | Autosomal recessive and dominant mutations causes deafness |
| Impg1 (ENSRNOG00000012479) | 8:85372670-85523184 | IMPG1 (ENSG00000112706) | 6:76687782-76839055 | Stargardt-like macular dystrophy |
| Htr1b (ENSRNOG00000013042) | 8:86700088-86701248 | HTR1B (ENSG00000135312) | 6:78228641-78229900 | A-161T locus, seems to impact HTR1B transcription, could play a role in suicide predisposition |
| LOC682486 (ENSRNOG00000023794) | 8:86996339-87135441 | AL591500.6 (ENSG00000214481) | 6:78457108-78689776 |  |
| ENSRNOG00000000896 (ENSRNOG00000000896) | 8:87915867-87916373 |  |  |  |
| Irak1bp1 (ENSRNOG00000008984) | 8:87951907-87973277 | IRAK1BP1 (ENSG00000146243) | 6:79633908-79665039 |  |
| RGD1564964_predicted (ENSRNOG00000008652) | 8:88011434-88060556 | PHIP (ENSG00000146247) | 6:79707008-79844708 |  |
| Hmgn3 (ENSRNOG00000031032) | 8:88169409-88179028 | HMGN3 (ENSG00000118418) | 6:79967685-80001125 |  |
| RGD1308555 (ENSRNOG00000009580) | 8:88464748-88522568 | C6orf152 (ENSG00000135338) | 6:80251427-80565440 | Leber Congenital Amaurosis (retinal blindness), autosomal recessive |
| LOC501026 (ENSRNOG00000009613) | 8:88608848-88654085 | SH3BGRL2 (ENSG00000198478) | 6:80397739-80470088 |  |
| RGD1561808_predicted (ENSRNOG00000030586) | 8:88819451-88821496 |  |  |  |
| RGD1561944_predicted (ENSRNOG00000001591) | 8:88843584-88844077 |  |  |  |
| Elovl4_predicted (ENSRNOG00000009773) | 8:88855623-88881703 | ELOVL4 (ENSG00000118402) | 6:80681255-80713941 | Deletion/frameshift mutation of gene causes Stargardt macular dystrophy |
| NP_001101642.1 (ENSRNOG00000029055) | 8:88882011-88944340 | TTK (ENSG00000112742) | 6:80771076-80808958 |  |
| Bckdhb (ENSRNOG00000009928) | 8:88997979-89191006 | BCKDHB (ENSG00000083123) | 6:80873083-81112706 | Various mutations in gene reported, cause Maple syrup urine disease |
| ENSRNOG00000037572 (ENSRNOG00000037572) | 8:89072597-89074004 |  |  |  |
| RGD1560917_predicted (ENSRNOG00000034128) | 8:89362012-89362741 | MRPL41 (ENSG00000182154) | 9:139566165-139566827 |  |
| NP_001100314.1 (ENSRNOG00000010240) | 8:90503082-90506227 | FAM46A (ENSG00000112773) | 6:82512166-82519210 | Inherited retina disease |
| Ibtk_predicted (ENSRNOG00000027728) | 8:90908796-90974009 | IBTK (ENSG00000005700) | 6:82936675-83014190 |  |
| ENSRNOG00000029843 (ENSRNOG00000029843) | 8:91023091-91024329 |  |  |  |
| LOC682994 (ENSRNOG00000029460) | 8:91028074-91028367 | FAM162A (ENSG00000114023) | 3:123585713-123611651 |  |
| Tpbg (ENSRNOG00000010694) | 8:91075960-91079329 | TPBG (ENSG00000146242) | 6:83130258-83133333 |  |

Supplementary table 1.
